# Development and validation of nomogram for predicting early recurrence after radical gastrectomy of gastric cancer

**DOI:** 10.1186/s12957-023-03294-1

**Published:** 2024-01-19

**Authors:** Mengxuan Cao, Can Hu, Siwei Pan, Yanqiang Zhang, Pengcheng Yu, Ruolan Zhang, Xiangdong Cheng, Zhiyuan Xu

**Affiliations:** 1 Department of Gastric Surgery, Zhejiang Cancer Hospital, Hangzhou Institute of Medicine (HIM), Chinese Academy of Sciences, Hangzhou, 310022 China; 2 Institutes of Basic Medicine and Cancer (IBMC), Chinese Academy of Sciences, Hangzhou, 310018 China; 3 Wenzhou Medical University, Wenzhou, 325035 China; 4 Department of Colorectal Surgery, Affiliated Jinhua Hospital, Zhejiang University School of Medicine, Jinhua, 321000 China

**Keywords:** Gastric cancer, Early recurrence, Nomogram, Prediction, Relapse pattern

## Abstract

**Background:**

After radical surgery, early detection of recurrence and metastasis is a crucial factor in enhancing the prognosis and survival of patients with gastric cancer (GC). Therefore, assessing the risk of recurrence in gastric cancer patients and determining the timing for postoperative recurrence is crucial.

**Methods:**

The clinicopathological data of 521 patients with recurrent gastric cancer, who underwent radical gastrectomy at Zhejiang Cancer Hospital between January 2010 and January 2017, were retrospectively analyzed. These patients were randomly divided into two groups: a training group (*n* = 365) and a validation group (*n* = 156). In the training set, patients were further categorized into early recurrence (*n* = 263) and late recurrence (*n* = 102) groups based on a 2-year boundary. Comparative analyses of clinicopathological features and prognoses were conducted between these two groups. Subsequently, a nomogram for predicting early recurrence was developed and validated.

**Results:**

In this study, the developed nomogram incorporated age, serous infiltration, lymph node metastasis, recurrence mode, and the tumour marker CA19-9. In the training cohort, the area under the curve (AUC value) was 0.739 (95% CI, 0.682–0.798), with a corresponding C-index of 0.739. This nomogram was subsequently validated in an independent validation cohort, yielding an AUC of 0.743 (95% CI, 0.652–0.833) and a C-index of 0.743. Furthermore, independent risk factors for prognosis were identified, including age, absence of postoperative chemotherapy, early recurrence, lymph node metastasis, abdominal metastasis, and vascular cancer embolus.

**Conclusion:**

Independent risk factors for gastric cancer recurrence following radical surgery were utilized to construct a nomogram for predicting early relapse. This nomogram effectively assesses the risk of recurrence, aids in treatment decision-making and follow-up planning in clinical settings, and demonstrated strong performance in the validation cohort.

**Supplementary Information:**

The online version contains supplementary material available at 10.1186/s12957-023-03294-1.

## Introduction

Gastric cancer (GC) ranks as the fifth most prevalent malignancy globally, with the highest incidence recorded in East Asia, and generally, it exhibits a higher prevalence in men than in women [1]. Although the worldwide incidence of gastric cancer has displayed a significant decline in recent years [2], it remains the third leading cause of cancer-related mortality [3]. With the advancement of medical technology and treatment model, the current treatment method for GC is the comprehensive management of surgery combined with chemotherapy. Combined treatment regimens have demonstrated a substantial enhancement in overall survival when compared to surgery alone [4, 5]. While most patients can achieve improved medical remission through individualized treatment, those who experience postoperative gastric cancer recurrence often face unsatisfactory therapeutic outcomes, with a notably reduced overall postoperative survival rate compared to patients without recurrence [6, 7]. Postoperative recurrence stands as a significant contributor to the dismal prognosis and heightened mortality in gastric cancer cases. In China, over 60% of patients with advanced gastric cancer encounter recurrence and metastasis following surgical intervention [8].

The recurrence patterns of gastric cancer are primarily categorized into abdominal metastasis, distant metastasis or hematogenous metastasis, and local recurrence, each exhibiting distinct recurrence timeframes [9, 10]. Most instances of postoperative gastric cancer recurrence occur within the first 2 years [11, 12], and carry a high short-term mortality rate [13]. Therefore, early diagnosis and treatment intervention for postoperative gastric cancer recurrence become pivotal for improving prognosis. Presently, the prognosis of gastric cancer patients primarily relies on the TNM staging system [14]. However, an increasing number of scholars advocate that a nomogram represents a more effective tool for predicting tumour progression and guiding clinical decision-making [15, 16]. Considering the challenging survival rates after gastric cancer recurrence, several foreign studies have explored risk factors for gastric cancer recurrence and the development of nomograms for predicting disease-free survival (DFS) [17–19]. For instance, Tonello et al. [17] established a nomogram to predict DFS, incorporating the Lauren classification and lymph node ratio (LNR). In a retrospective study by Spolverato et al. [18], it was determined that tumour site, depth of invasion, LNR, and lymphovascular invasion (LVI) were independent risk factors for recurrence. However, most of the predictive studies originate from Europe and the Americas, with limited research on the development of nomograms for predicting early recurrence in China.

In this study, our objective is to construct an effective prediction model for early gastric cancer recurrence following surgery and investigate the factors influencing the prognosis of patients with recurrent gastric cancer.

## Methods

### Patients

Between January 2010 and January 2017, a total of 4,308 patients underwent curative gastrectomy at Zhejiang Cancer Hospital, and follow-up continued until May 2022. Out of these, 606 patients experienced recurrence after surgery, 35 patients were lost to follow-up, and an additional 50 patients had incomplete data (Supplementary Fig. S1). Therefore, the study included 521 patients who experienced postoperative recurrence. To ensure the reliability of the study, the research team randomly divided the patient cohort into a training set and a validation set in a 7:3 ratio. This allocation resulted in 365 cases being assigned to the training cohort and 156 cases to the validation cohort. Inclusion criteria comprised the following: (1) Confirmation of primary gastric adenocarcinoma through postoperative pathological examination; (2) Radical resection following R0 resection of gastric cancer with negative margins both above and below the tumour site; (3) Postoperative recurrence and metastasis; (4) Lymph node dissection classified as D2 or higher; (5) Absence of recurrence or death within 1 month following radical surgery; (6) Availability of complete clinical treatment records and comprehensive follow-up data, which were maintained through regular outpatient follow-up and telephone follow-up. The demographic and clinicopathological data of the two cohorts are presented in Supplementary Table S1.

### Data management

According to the DFS criteria, the 365 patients in the training set were categorized into two groups: the early recurrence group (*n* = 263, DFS ≤ 24 months) and the late recurrence group (*n* = 102, DFS > 24 months). We collected data across various clinical categories, including: sex, BMI (≥ 28, < 28), overall survival (OS), age (> 60 years old, ≤ 60 years old), pTNM staging [refer to AJCC/UICC 8th Edition TNM staging criteria for gastric cancer], serosa infiltration, primary site (cardia, body, antrum, whole), degree of tissue differentiation (low-moderate, medium–high), tumour length (≥ 5 cm, < 5 cm), pN staging (N0/1/2, N3), histological type (adenocarcinoma, signet-ring cell carcinoma), nerve invasion, vascular cancer thrombus, recurrence mode (liver, abdominal, retroperitoneal lymph node, ovarian, loco-regional, others), chemotherapy regimen (preoperative neoadjuvant chemotherapy, postoperative adjuvant chemotherapy), HER2 expression and the oncology examination markers (CEA, AFP, CA19-9, CA125, CA242, CA72-4) (Table 1).

**Table 1:** Univariate and multivariate analysis of early recurrence in patients with gastric cancer in train cohort

Variables	Early recurrence	Late recurrence	Univariate			Multivariate		
	***N* = 263**	***N* = 102**	**OR**	**95%CI**	***P***	**OR**	**95%CI**	***P***
**Age**								
≤ 60	143	69	Ref			Ref		
> 60	120	33	1.755	1.085 ~ 2.837	**0.022**	1.789	1.043 ~ 3.068	**0.035**
**Sex**								
Female	80	31	Ref					
Male	183	71	1.001	0.609 ~ 1.646	0.996			
**Tumour length**								
< 5cm	107	50	Ref					
≥ 5cm	156	52	1.402	0.852 ~ 2.220	0.150			
**Nerve invasion**								
Positive	86	33	Ref					
Negative	177	69	0.984	0.604 ~ 1.604	0.949			
**Serosa infiltration**								
Negative	20	21	Ref			Ref		
Positive	243	81	3.150	1.625 ~ 6.107	**0.001**	2.293	1.093 ~ 4.811	**0.028**
**Neoadjuvant chemotherapy**								
Positive	39	22	Ref					
Negative	224	80	1.169	0.832 ~ 1.643	0.368			
**Postoperative chemotherapy**								
Positive	213	73	Ref					
Negative	50	29	1.526	0.899 ~ 1.856	0.085			
**Histological type**								
Adenocarcinoma	172	46	Ref					
SRCC^a^	124	23	1.234	0.979 ~ 1.556	0.075			
**Lymph node staging**								
N0/1/2	151	73	Ref			Ref		
N3	112	29	1.867	1.138 ~ 3.062	**0.013**	2.162	1.225 ~ 3.815	**0.008**
**Differentiated degree**								
Medium–High	31	18	Ref					
Low-Medium	232	84	1.604	0.852 ~ 3.018	0.143			
**Recurrence mode**								
Liver	76	11	5.873	2.691 ~ 12.814	**0.000**	7.031	3.048 ~ 16.218	**0.000**
Abdominal	92	31	2.523	1.368 ~ 4.652	**0.003**	2.713	1.420 ~ 5.184	**0.003**
Retroperitoneal LN	20	3	5.667	1.549 ~ 20.725	**0.009**	5.696	1.498 ~ 21.658	**0.033**
Ovary	13	7	1.579	0.566 ~ 4.405	0.383	1.976	0.661 ~ 5.907	0.223
Loco-regional	22	16	1.169	0.531 ~ 2.575	0.699	1.655	0.704 ~ 3.894	0.248
Else	40	34	Ref			Ref		
**CA19-9**								
Normal	172	81	Ref	1.186 ~ 3.513	**0.010**	Ref	1.096 ~ 3.542	**0.023**
Abnormal	91	21	2.041			1.970		
**CA72-4**								
Normal	179	81	Ref			Ref		
Abnormal	84	21	1.810	1.049 ~ 3.123	**0.033**	1.352	0.743 ~ 2.462	0.324

^a^*SRCC* Signet ring cell carcinoma. Data from Zhejiang Cancer Hospital. Reference range: CA19-9>37U/ml, CA72-4>6.7U/ml

In particular, Signet-ring cell carcinoma (SRCC) in gastric cancer tissue samples was defined as having more than half of the cells exhibiting signet-ring cell characteristics. Abdominal metastasis was characterized by the presence of abdominal wall masses, peritoneal nodules, or positive ascites cytology. Retroperitoneal lymph node metastases were considered positive when lymph node metastases were found in group 16 during postoperative examination. Loco-regional recurrence encompassed instances of residual stomach and anastomotic site recurrence, along with regional lymph node metastasis. Brain metastases, lung metastases, renal metastases, and pancreas metastases were categorized as other relapses. The recurrence mode is based on the site of first recurrence. Serosa infiltration was defined as stage T3/4. Neoadjuvant chemotherapy refers to adjuvant chemotherapy performed before surgery. Postoperative chemotherapy refers to adjuvant chemotherapy administered between the surgery and the occurrence of recurrence. The primary adjuvant chemotherapy regimens included SOX, XELOX, FOLFOX, FLOT, and others. All tumour markers are detected before surgery. Tumour markers were considered positive if they exceeded the normal range, with reference ranges as follows: CEA > 5 ng/ml, CA19-9 > 37U/ml, CA125 > 35U/ml, AFP > 25 ng/ml, CA242 > 20U/ml, CA72-4 > 6.7U/ml. Disease-free survival (DFS) and overall survival (OS) calculations commenced from the date of radical operation.

This study was conducted in compliance with the ethical principles for medical research outlined in the World Medical Association’s Declaration of Helsinki. Furthermore, the research protocol received approval from the Research Ethics Committee of Zhejiang Cancer Hospital (IRB-2022–371).

### Follow-up plan

We defined the recurrence time (≤ 2 years) as early recurrence. Following surgery, all patients underwent periodic re-examinations. Specifically, for the first 2 years after surgery, re-examinations were conducted every 3 months, and from 2 to 5 years post-surgery, they were scheduled every 3 to 6 months. These examinations included physical assessments, routine blood tests, gastroscopy, ultrasound, chest X-rays, computed tomography (CT), magnetic resonance imaging (MRI) and tissue puncture biopsy. In cases where needed, laparoscopic exploration was employed to ascertain clear evidence of gastric cancer recurrence.

### Statistical analysis

We used SPSS 25.0 for statistical analysis, employing both univariate and multivariate logistic regression to assess various clinical variables. To estimate overall survival post-recurrence, we applied the Kaplan–Meier method and established a multivariate Cox risk regression model to evaluate clinical factors affecting postoperative survival. Results with a significance level of *P* < 0.05 were considered statistically significant. For constructing a nomogram based on the multivariate analysis results, we used the RMS software package. The nomogram’s accuracy was assessed through validation, and its performance was evaluated using Harrell’s C-index and the AUC value. The C-index ranges from 0.5 to 1.0, where 0.5 suggests random chance and 1.0 indicates the model’s precise ability to differentiate results. The AUC ranges from 0.5 to 1.0, with values between 0.5 and 0.7 indicating low accuracy, 0.7 to 0.9 suggesting moderate accuracy, and values above 0.9 representing high accuracy. Subsequently, we verify it with calibration curves and decision curves.

## Results

### The time and mode of early and late recurrence of gastric cancer

**Fig. 1:**
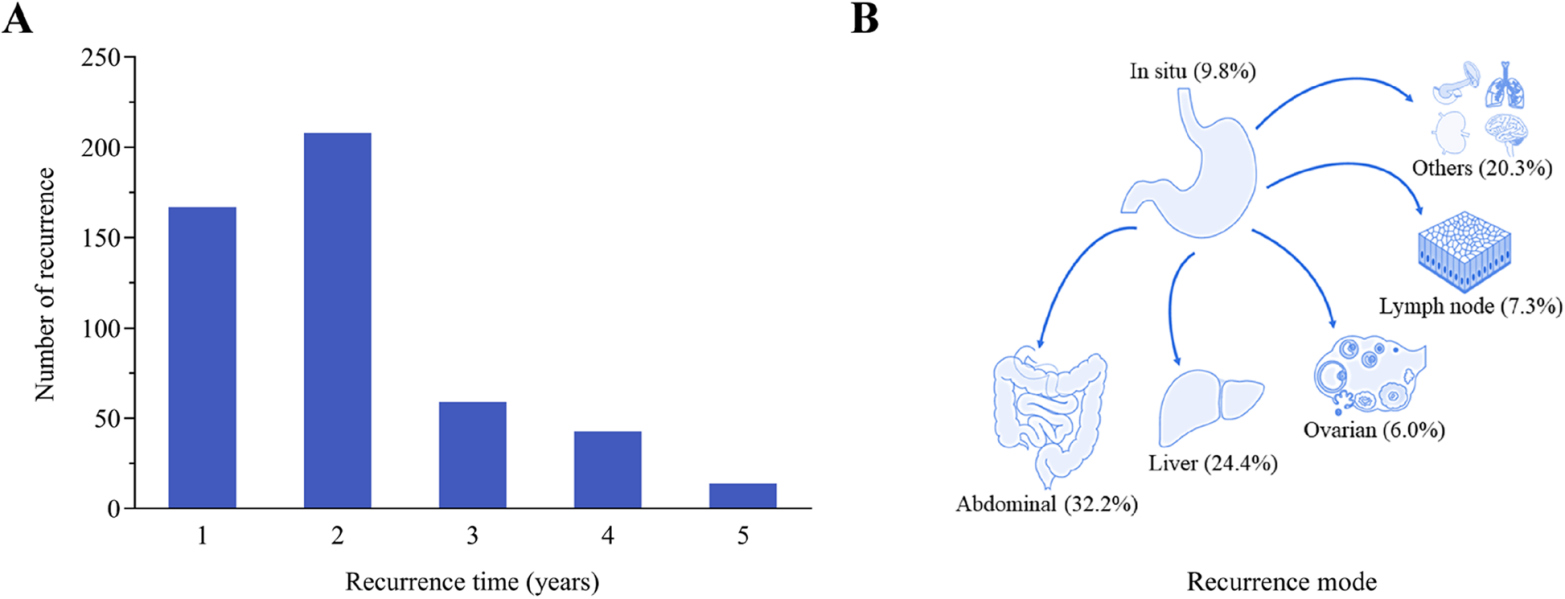
Recurrence regularity of gastric cancer patients. Recurrence cases (**A**) and recurrence modes (**B**) within 5 years in all patients with gastric cancer

Among the 521 postoperative patients included in this study, there were 365 males and 156 females. The overall median recurrence time was 16 months (IQR, 9–27 months). The mean recurrence time for patients was 22.23 months (95% CI: 20.51–23.96 months). Postoperative recurrence mainly occurred within 2 years. The recurrence rates at 1-, 2-, 3-, 4-, and 5- years after surgery were 32.1% (167/521), 72.0% (375/521), 83.3% (434/521), 91.5% (477/521), and 94.2% (491/521), respectively (Fig. 1A). Among them, there were 375 patients with early recurrence; the median recurrence time was 12 months (IQR, 8–18 months). Additionally, there were 146 patients with late recurrence, and the median recurrence time was 40 months (IQR, 32–55 months).

In this study, abdominal metastasis (*n* = 168, 32.2%) was the most common mode of recurrence, followed by liver metastasis (*n* = 127, 24.4%). Patients with ovarian metastases (*n* = 31, 6.0%) had the least recurrence. Additionally, the remaining metastatic sites included retroperitoneal lymph node metastasis (*n* = 38, 7.3%), in situ recurrence (*n* = 51, 9.8%), and others (*n* = 106, 20.3%) (Fig. 1B).

### Risk factors for early recurrence of gastric cancer

A total of 365 patients in the training cohort were classified into early and late recurrence groups, with the division occurring at the 2-year mark. Univariate and multivariate analyses were conducted to explore the risk factors that could influence early gastric cancer recurrence. Age, serosa infiltration, lymph node metastasis, mode of recurrence, and tumour markers CA19-9 and CA72-4 were found to be closely associated with early gastric cancer recurrence (Table 1). All covariables were included in a multivariate logistic regression analysis to account for the influence of these variables. Consequently, the multivariate logistic regression model revealed that age (OR = 1.789, *P* < 0.05), serosa infiltration (OR = 2.293, *P* < 0.05), lymph node metastasis (OR = 2.162, *P* < 0.05), liver metastasis (OR = 7.031, *P* < 0.05), abdominal metastasis (OR = 2.731, *P* < 0.05), retroperitoneal lymph node metastasis (OR = 5.696, *P* < 0.05), and the tumour marker CA19-9 (OR = 1.970, *P* < 0.05) were independent risk factors for early recurrence (Table 1).

### Risk factors for survival of gastric cancer recurrence

The overall median postoperative survival for all 521 patients was 28 months (IQR, 17–50 months), and the average survival time was 37.46 months (95% CI: 34.92–40.00 months). The survival rates at 1-, 3-, and 5- years after surgery were 87.5% (456/521), 38.6% (201/521), and 17.7% (92/521), respectively.

**Fig. 2:**
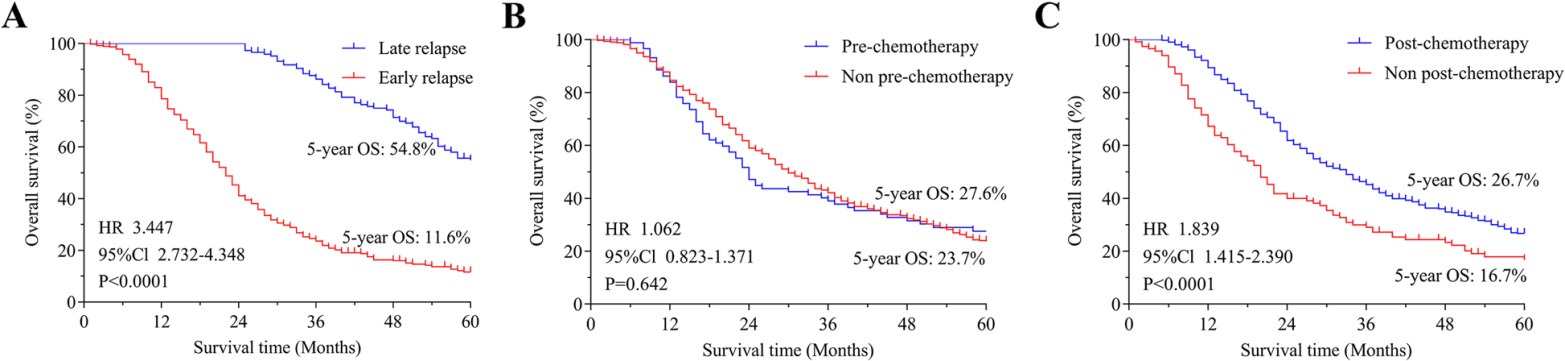
Cumulative survival curves after surgery for different risk factors among all recurrent patients. Survival curves comparing the early recurrence group with the late recurrence group (**A**). Survival curves comparing groups that received preoperative neoadjuvant chemotherapy with those that did not (**B**). Survival curves comparing groups that received postoperative adjuvant chemotherapy with those that did not (**C**)

Furthermore, the survival analysis of all patients included in this study revealed that age > 60 (HR = 1.357, *P* < 0.05), early recurrence (HR = 3.265, *P* < 0.05), vascular tumour thrombus (HR = 1.572, *P* < 0.05), non-postoperative chemotherapy (HR = 2.069, *P* < 0.05), lymph node staging (HR = 1.409, *P* < 0.05), and abdominal metastasis (HR = 1.513, *P* < 0.05) were independent risk factors affecting the survival of patients with postoperative recurrence (Table 2). Among these factors, early recurrence was identified as the most significant risk factor influencing postoperative survival. This observation is supported by the survival curve depicted in Fig. 2A, indicating that the survival of patients with late recurrence was significantly better than that of patients with early recurrence (HR = 3.447, *P* < 0.05).

**Table 2:** Univariate and multivariate analysis of OS in postoperative patients with gastric cancer

Variables	Univariate		Multivariate		
	**HR**	***P***	**HR**	**95%CI**	***P***
**Age**					
≤ 60	Ref		Ref		
> 60	1.416	**0.000**	1.357	1.099 ~ 1.675	**0.005**
**Tumour length**					
< 5cm	Ref		Ref		
≥ 5cm	1.380	**0.001**	0.844	0.680 ~ 1.048	0.124
**Vascular tumour thrombus**					
Negative	Ref		Ref		
Positive	1.741	**0.000**	1.572	1.251 ~ 1.697	**0.000**
**Nerve infiltration**					
Negative	Ref		Ref		
Positive	1.475	**0.000**	1.008	0.788 ~ 1.291	0.948
**Serosa infiltration**					
Negative	Ref		Ref		
Positive	2.660	**0.000**	1.413	0.898 ~ 2.224	0.135
**Neoadjuvant chemotherapy**					
Positive	Ref				
Negative	1.062	0.642			
**Postoperative chemotherapy**					
Positive	Ref		Ref		
Negative	1.659	**0.000**	2.069	1.629 ~ 2.629	**0.000**
**Differentiation**					
Medium–High	Ref		Ref		
Low-Medium	1.635	**0.001**	1.215	0.902 ~ 1.638	0.201
**Lymph node staging**					
N0/1/2	Ref		Ref		
N3	1.784	**0.000**	1.409	1.130 ~ 1.756	**0.002**
**pTNM stage**^**a**^					
I	Ref		Ref		
II	2.001	**0.010**	1.705	0.942 ~ 3.086	0.078
III	2.839	**0.000**	1.793	0.977 ~ 3.291	0.059
**Recurrence mode**					
Liver	1.458	**0.012**	1.278	0.938 ~ 1.740	0.120
Abdominal	1.669	**0.000**	1.573	1.142 ~ 2.006	**0.004**
Retroperitoneal LN	1.592	**0.030**	1.022	0.666 ~ 1.570	0.920
Ovary	0.855	0.532	0.739	0.449 ~ 1.218	0.236
Loco-regional	0.938	0.736	0.764	0.520 ~ 1.123	0.170
Else	Ref		Ref		
**Early recurrence**					
No	Ref		Ref		
Yes	3.447	**0.000**	3.265	2.535 ~ 4.206	**0.000**
**CA19-9**					
Normal	Ref		Ref		
Abnormal	1.395	**0.002**	1.240	0.961 ~ 1.601	0.099
**CA242**					
Normal	Ref		Ref		
Abnormal	1.365	**0.014**	0.962	0.708 ~ 1.307	0.803
**CA72-4**					
Normal	Ref		Ref		
Abnormal	1.584	**0.000**	1.165	0.930 ~ 1.459	0.184

^a^pTNM stage: Pathological tumour, node, metastasis staging system, AJCC 8th. Data from Zhejiang Cancer Hospital. Reference range: CA19-9 > 37U/ml, CA242 > 20U/ml, CA72-4 > 6.7U/ml

Additionally, an analysis was conducted to examine the relationship between adjuvant chemotherapy and postoperative survival. The results revealed that there was no significant association between preoperative neoadjuvant chemotherapy and postoperative survival (Fig. 2B). On the other hand, patients who underwent postoperative adjuvant chemotherapy exhibited significantly better survival rates compared to those who did not receive chemotherapy (HR = 1.839, *P* < 0.05) (Fig. 2C). These findings indicate a clear positive effect of postoperative adjuvant chemotherapy on postoperative survival.

### Subgroup survival analysis of postoperative adjuvant chemotherapy

**Fig. 3:**
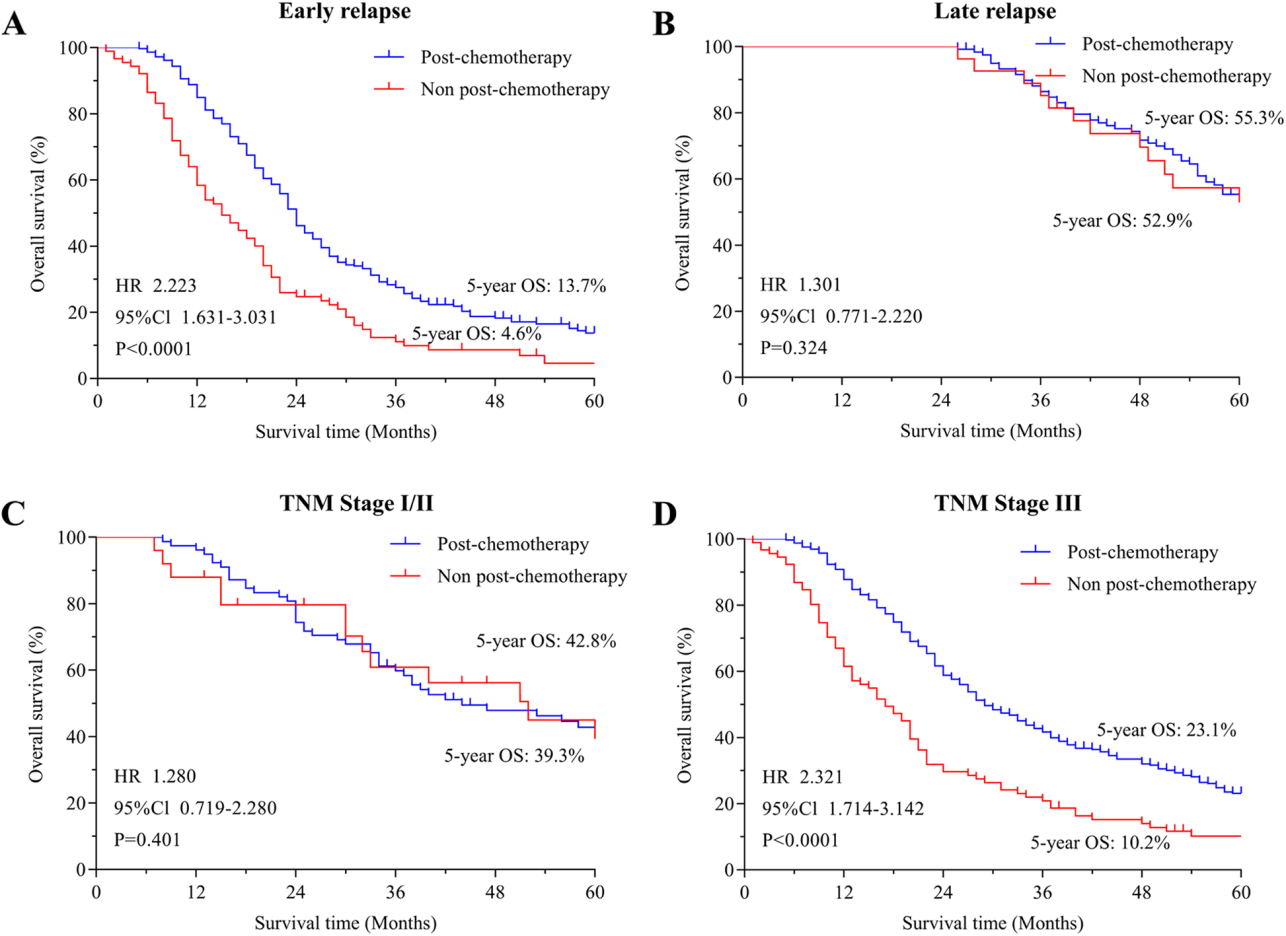
Subgroup survival analysis of postoperative adjuvant chemotherapy. Prognostic differences between early relapse (**A**) and late relapse (**B**) patients with adjuvant chemotherapy after surgery. Postoperative adjuvant chemotherapy in patients with TNM stage I/II (**C**) and TNM stage III (**D**)

Further subgroup analysis was conducted regarding the impact of postoperative adjuvant chemotherapy on early and late recurrence patients. The analysis demonstrated a substantial survival benefit in early recurrence patients (HR = 2.223, *P* < 0.05) (Fig. 3A), whereas there was no significant difference observed in late recurrence patients (Fig. 3B).

Moreover, the study classified patients into two groups based on TNM staging (I/II, III). Figures 3C and 3D illustrate the differential impact of postoperative adjuvant chemotherapy on patients with different TNM stages. Remarkably, only stage III gastric cancer patients experienced a significant survival benefit with postoperative adjuvant chemotherapy (HR = 2.321, *P* < 0.05). These results suggest that the positive survival effects of postoperative adjuvant chemotherapy are more significant in early recurrent cases and in patients with stage III gastric cancer.

### Development and validation of model for predicting early recurrence

**Fig. 4:**
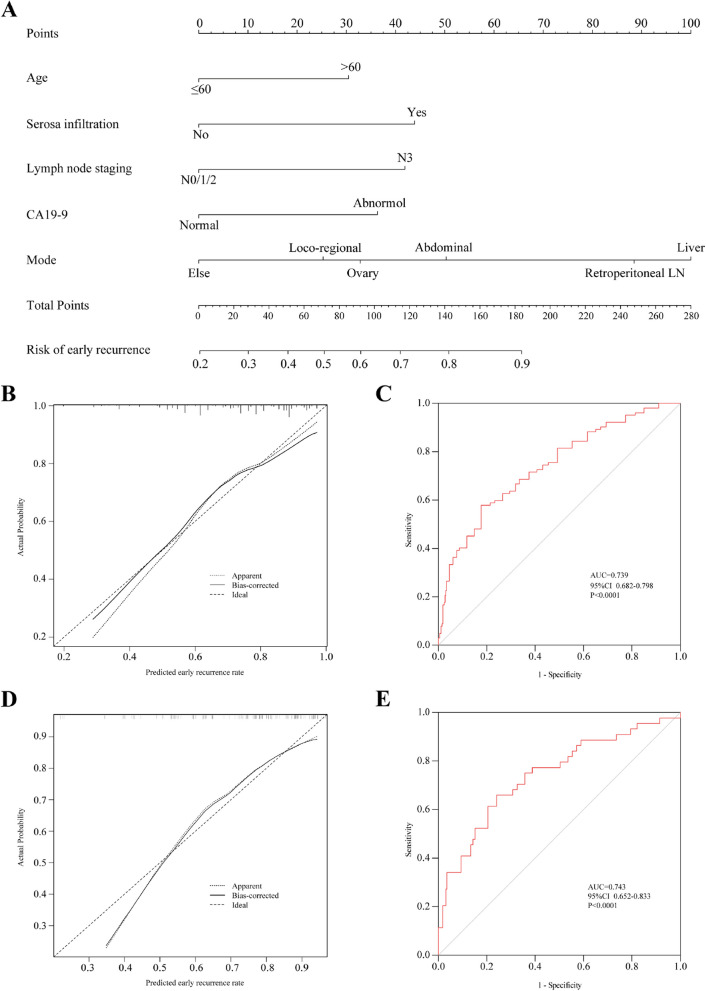
Development and validation of prediction model. Nomogram for predicting early postoperative recurrence in patients with gastric cancer (**A**). Calibration curves of nomogram in training cohort (**B**) and verification cohort (**C**). The diagonal line represents the performance of ideal nomogram, and the solid line represents the consistency between the built nomogram and the actual nomogram. ROC curves of nomogram in the training cohort (**D**) and in the verification cohort (**E**). The area below the red line (AUC) represents the performance of nomogram

A clinical nomogram was developed based on independent predictors of early recurrence to estimate the risk of early postoperative recurrence in gastric cancer patients (Fig. 4A). The nomogram included age, serous infiltration, lymph node metastasis, recurrence mode, and the tumour marker CA19-9 as predictors. In the training cohort, Fig. 4B displays the calibration curve used for predicting the probability of early recurrence. The results indicated that the nomogram’s predictions aligned well with the actual observations, with a mean absolute error (MAE) of 0.027. The area under the curve (AUC) for this prediction model was 0.739 (95% CI: 0.682–0.798) (Fig. 4C), and the C-index for predicting early recurrence was 0.739.

### Validation the predictive accuracy of nomogram for early recurrence

To assess the suitability of the model, a validation cohort consisting of 165 patients from the same center was used. Independent risk factors included in the nomogram were evaluated in the validation cohort. Figure 4D indicates good consistency of the nomogram calibration curves for predicting the risk of early recurrence, with a MAE of 0.027. The AUC for this prediction model in the validation cohort was 0.743 (95% CI: 0.652–0.833) (Fig. 4E), and the C-index for predicting early recurrence in the validation cohort was 0.743.

### Decision curve analysis

**Fig. 5:**
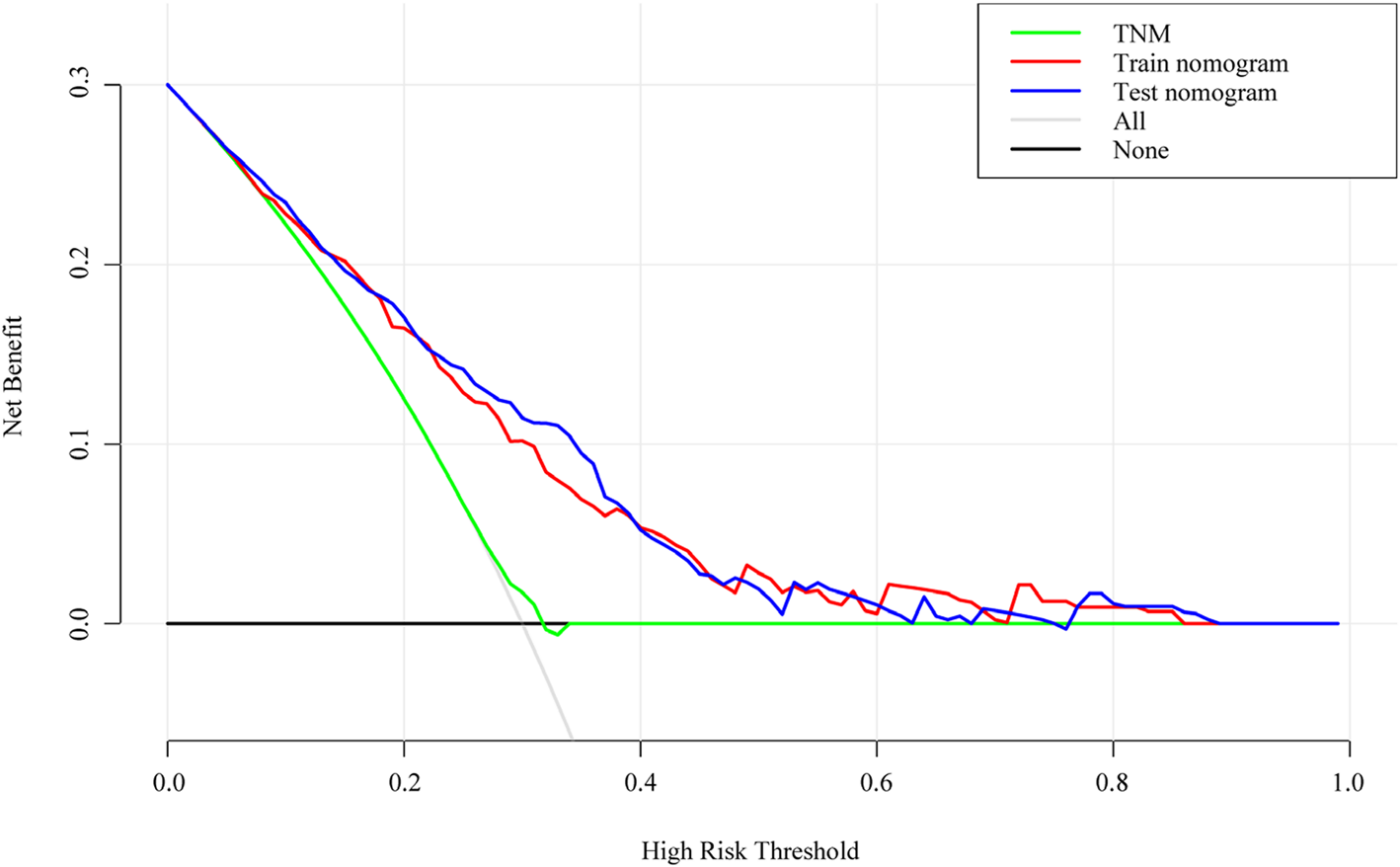
Decision curve analysis for prediction of early recurrence. The black line represents the net benefit of none of the patients receiving general treatment interventions; the gray line represents the net benefit of patients receiving general treatment interventions; the green line represents the net benefit for patients receiving pTNM staging interventions; the red and blue lines represent the net benefit for patients receiving nomogram interventions in the training cohort and validation cohort, respectively. The red and blue lines is above the rest of lines, indicating that the nomogram provides clinical benefit

Figure 5 displays a decision curve analysis comparing the developed nomogram with the TNM staging system. The analysis shows that both the nomogram and TNM staging are beneficial for predicting early recurrence when the patient’s probability exceeds 30%. The nomogram outperforms the 8th AJCC-TNM system in both cohorts, indicating its superior predictive ability for early recurrence in gastric cancer patients.

## Discussion

While the incidence of gastric cancer has shown a decreasing trend in China over the years, it remains a prevalent disease impacting public health [20]. Recurrence and metastasis following radical gastrectomy are critical factors contributing to the unfavorable prognosis of patients [6]. Notably, in China, limited attention has been devoted to the timing of gastric cancer recurrence, and available recurrence data often rely on small sample sizes [21, 22]. Hence, this study aims to investigate the recurrence patterns of gastric cancer in our country and establish a risk model for early recurrence prediction. The resulting nomogram not only identifies high-risk groups for gastric cancer recurrence but also offers valuable guidance to clinicians in devising effective follow-up strategies.

Comparing our patient data with those of the United States and South Korea, we observed lower rates of loco-regional recurrence (25.7% ~ 44.1% vs. 9.0%) [9, 23]. In a multicenter retrospective study conducted by Dan et al. [8], peritoneal metastasis rates in China were recorded at 13.7%. In contrast, our study revealed a significantly higher rate of abdominal metastases (32.2%), aligning more closely with the rates observed in South Korea and the United States (26.0% ~ 45.9%) [9, 23, 24]. It is worth noting that a substantial portion of Dan’s study data was from 15 years ago, possibly due to limited imaging sensitivity and the infrequent use of laparoscopic exploration at that time [25]. Our study, in contrast, predominantly comprises cases from the last decade, thus offering a more contemporary depiction of the gastric cancer recurrence landscape in our country. Previous research has established that early recurrence primarily involves hematogenous metastasis, whereas late recurrence predominantly manifests as loco-regional recurrence [9, 24, 26]. Our study results corroborate this finding, with the abdominal cavity (*n* = 168, 32.2%) emerging as the primary site for postoperative gastric cancer metastasis. Specifically, early recurrence is primarily associated with abdominal metastasis (*n* = 127, 33.9%), while late recurrence is more frequently observed in other anatomical sites (*n* = 49, 33.5%). Furthermore, our constructed nomogram highlights the significance of metastatic location as one of the key predictive factors. Independent risk factors for early recurrence include abdominal metastasis, liver metastasis, and retroperitoneal lymph node metastasis, with liver metastasis bearing the highest risk coefficient among them.

Numerous studies have consistently demonstrated that the lymph node metastasis as an independent predictor of early recurrence [11, 22, 23, 27]. Our research led us to the conclusion that the risk of early recurrence significantly escalates when postoperative lymph node pathological staging reaches N3. In line with our findings, Kim et al. [28] identified age, LNR, and serous infiltration as potential predictors of recurrence. However, our prediction model surpasses their established nomogram in performance for predicting DFS (CI 0.74 vs. 0.71) and accounts for a broader range of influencing factors. Furthermore, our study supports the notion that serous infiltration is a pivotal risk factor for postoperative peritoneal recurrence and metastasis in patients with gastric cancer, a finding consistent with several previous studies [22, 27]. For patients with serous infiltration, multiple studies have reported that combining D2 radical surgery with hyperthermic intraperitoneal chemotherapy effectively reduces the incidence of postoperative peritoneal metastasis without causing a significant increase in perioperative complications [29, 30]. Nevertheless, it’s worth noting that there is currently no universally accepted standard regarding population selection, treatment plans, drug dosages, perfusion frequencies, temperature control, and other aspects of prophylactic hyperthermic intraperitoneal chemotherapy. Consequently, further clinical trials are essential to provide definitive answers to these critical questions. Previous research has stressed the significance of tumour markers associated with gastric cancer in the monitoring of recurrence [31]. Hence, this study took into account the impact of preoperative tumour markers. The developed nomogram integrates the tumour marker CA19-9, underscoring its predictive value in identifying high-risk recurrence patients. Consequently, we advocate the routine assessment of preoperative tumour markers in individuals diagnosed with gastric cancer.

Furthermore, exploring factors influencing OS in GC patients is crucial. Our research underscores that the most critical factor affecting postoperative survival rates in gastric cancer recurrence patients is early relapse. This viewpoint aligns with the findings of Kodera et al. [32], who assessed the impact of routine follow-up on postoperative survival. Their study indicated that while close monitoring aids in early recurrence detection and significantly improves survival rates once recurrence is identified, it doesn’t bring substantial overall benefits to postoperative survival. This is primarily due to early relapses and a shorter DFS time. Many previous studies have demonstrated the impact of neoadjuvant and adjuvant chemotherapy on recurrence and survival in gastric cancer patients [33, 34]. However, in contrast to previous reports, we did not find an association between chemotherapy and DFS. In a multifactor analysis, neoadjuvant chemotherapy did not significantly impact postoperative OS, but adjuvant chemotherapy showed significant benefits in improving OS. Subsequently, we obtained some interesting results in a subgroup survival analysis of postoperative adjuvant chemotherapy. Patients experiencing early relapse may have better survival or longer survival periods when receiving adjuvant chemotherapy. Regarding TNM staging, stage III patients showed a more significant improvement in prognosis with postoperative adjuvant chemotherapy. For patients with late relapses and stage I/II patients, the presence or absence of adjuvant chemotherapy did not show a significant difference in prognosis. These findings suggest that in the advanced disease stage, adjuvant chemotherapy may play a more critical role in disease management, emphasizing the importance of postoperative adjuvant chemotherapy in advanced disease. Currently, there have been many large clinical studies investigating the ability of postoperative adjuvant chemotherapy to extend the survival period of late-stage patients [35, 36]. Our study included a larger number of patients and considered the influence of other clinical and pathological factors, providing valuable insights. Furthermore, among various modes of recurrence, peritoneal metastasis demonstrated poorer postoperative OS compared to other recurrence patterns, as previously reported [37]. Peritoneal metastasis in gastric cancer is typically considered an advanced-stage condition often accompanied by complications like ascites, intestinal adhesions, and even obstructions, significantly impacting patients’ quality of life. Our study results serve as a warning to clinicians for preventing peritoneal recurrence and guiding subsequent treatments.

The eighth edition of the TNM classification remains pivotal for postoperative treatment and follow-up planning [14]. However, a growing body of research emphasizes alternative factors and tools for predicting postoperative recurrence risk and improving survival [12, 17, 19]. Our carefully crafted clinical prediction model encompasses a comprehensive set of factors and has shown robust performance in subsequent validation. In terms of clinical decision curve results, when compared to the traditional TNM staging system, our predictive model serves as a promising resource that can enhance informed decision-making in clinical practice and patient care. Its ability to integrate other factors and demonstrate robust performance in validation underscores its potential significance in optimizing patient prognosis.

Nevertheless, there are some limitations that should be noted. Firstly, it’s based on looking back at past data, with some patient data missing and possible biases in the way patients were selected to participate in the study. Secondly, this research only used data from one hospital, so the people in the study might not be very diverse. Lastly, while this study analyzed factors affecting OS in GC recurrence patients and considered adjuvant chemotherapy, it did not create a tool to predict OS after radical gastrectomy.

## Conclusion

The nomogram in our study can accurately predict the early postoperative recurrence rate of gastric cancer patients, and identify those patients at high risk of postoperative early recurrence, potentially promoting highly tailored patient management. This study not only improve people’s understanding of early recurrence of gastric cancer in Chinese or Asian populations, but also has certain guiding significance for the first line clinicians to make treatment plan and follow-up plan.

### Supplementary Information

**Additional file 1.**

## Data Availability

Due to the privacy of patients, the data related to patients cannot be available for public access but can be obtained from the corresponding author on reasonable request approved by the institutional review board of all enrolled centers.

## Abbreviations

GC: Gastric Cancer
TNM: Tumour Node Metastasis
OS: Overall Survival
DFS: Disease Free Survival
IQR: Inter Quartile Range
SRCC: Signet ring cell carcinoma
R0: Radical Resection
LVI: Lymphovascular Invasion
MAE: Mean Absolute Error
LNR: Lymph Node Ratio
BMI: Body Mass Index
CT: Computed Tomography
MRI: Magnetic Resonance Imaging
ROC: Receiver Operating Characteristic
AUC: Area Under Curve
OR: Odd Radio
HR: Hazard Ratio
RMS: Recession Modeling Strategy
