# Supplementary material for: Development and validation of nomogram for predicting early recurrence after radical gastrectomy of gastric cancer
Source: World J Surg Oncol. 2024 Jan 19;22:21. doi: 10.1186/s12957-023-03294-1 (PMC10797937; doi:10.1186/s12957-023-03294-1)
Supplement: Supplementary file 1 — Additional file 1. [file 12957_2023_3294_MOESM1_ESM.docx]

**Supplementary Table S1**

Clinicopathological data of 521 patients with recurrent gastric cancer after radical resection.

| **Variables** | **Train cohort** |  | **Validation cohort** |
| --- | --- | --- | --- |
|  | **N=365 (%)** |  | **N=156 (%)** |
| **Sex**  Male  Female | 254(69.6%)  111(30.4%) |  | 111(71.2%)  45(28.8%) |
| **Age**  >60  ≤60 | 153(41.9%)  212(58.1%) |  | 69(44.2%)  87(55.8%) |
| **BMI**  >28  ≤28 | 16(4.4%)  349(95.6%) |  | 4(2.6%)  152(97.4%) |
| **Tumour length**  ≥5cm  <5cm | 208(57.0%)  157(43.0%) |  | 86(55.1%)  70(44.9%) |
| **Histological type**  Adenocarcinoma  SRCC | 218(59.7%)  147(40.3%) |  | 106(67.9%)  50(32.1%) |
| **Differentiated degree**  Low-Medium  Medium-High | 316(86.6%)  49(13.4%) |  | 132(84.6%)  24(15.4%) |
| **Primary site**  Cardia  Body  Antrum  Whole | 105(28.8%)  86(23.5%)  166(45.5%)  8(2.2%) |  | 41(26.3%)  39(25.0%)  71(45.5%)  5(3.2%) |
| **Recurrence mode**  Liver  Abdominal  Retroperitoneal LN  Ovary  Loco-regional  Else | 87(23.8%)  123(33.7%)  23(6.3%)  20(5.5%)  38(10.4%)  74(20.3%) |  | 40(25.6%)  45(28.9%)  15(9.6%)  11(7.1%)  13(8.3%)  32(20.5%) |
| **Vascular tumour thrombus**  Positive  Negative | 225(61.6%)  140(38.4%) |  | 87(55.8%)  69(44.2%) |
| **Nerve invasion**  Positive  Negative | 246(67.4%)  119(32.6%) |  | 112(71.8%)  44(28.2%) |
| **Serosa infiltration**  Positive  Negative | 324(88.8%)  41(11.2%) |  | 131(84.0%)  25(16.0%) |
| **Neoadjuvant chemotherapy**  Positive  Negative | 61(16.7%)  304(83.3%) |  | 26(16.7%)  130(83.3%) |
| **Postoperative chemotherapy**  Positive  Negative | 286(78.4%)  79(21.6%) |  | 119(76.3%)  37(23.7%) |
| **Lymph node staging**  N0/1/2  N3 | 224(61.4%)  141(38.6%) |  | 56(35.9%)  100(64.1%) |
| **pTNM stage**  I  II  III | 24(6.6%)  45(12.3%)  296(81.1%) |  | 10(6.4%)  24(15.4%)  122(78.2%) |
| **CEA**  Normal  Abnormal | 271(74.2%)  94(25.8%) |  | 126(80.8%)  30(19.2%) |
| **CA19-9**  Normal  Abnormal | 253(69.3%)  112(30.7%) |  | 118(75.6%)  38(24.4%) |
| **CA125**  Normal  Abnormal | 327(89.6%)  38(10.4%) |  | 146(93.6%)  10(6.4%) |
| **AFP**  Normal  Abnormal | 349(95.6%)  16(4.4%) |  | 150(96.2%)  6(3.8%) |
| **CA242**  Normal  Abnormal | 297(81.4%)  68(18.6%) |  | 135(86.5%)  21(13.5%) |
| **CA72-4**  Normal  Abnormal | 260(71.2%)  105(28.8%) |  | 118(75.6%)  38(24.4%) |
| **HER2**  0/1+/2+  3+ | 347(95.1%)  18(4.9%) |  | 145(92.9%)  11(7.1%) |

*pTNM stage: Pathological tumour, node, metastasis staging system, AJCC 8th. pN-stage: pathological nodal stage. Data from Zhejiang Cancer Hospital. Reference range: CEA>5ng/ml, CA19-9>37U/ml, CA125>35U/ml, AFP>25ng/ml, CA242>20U/ml, CA72-4>6.7U/ml.

**
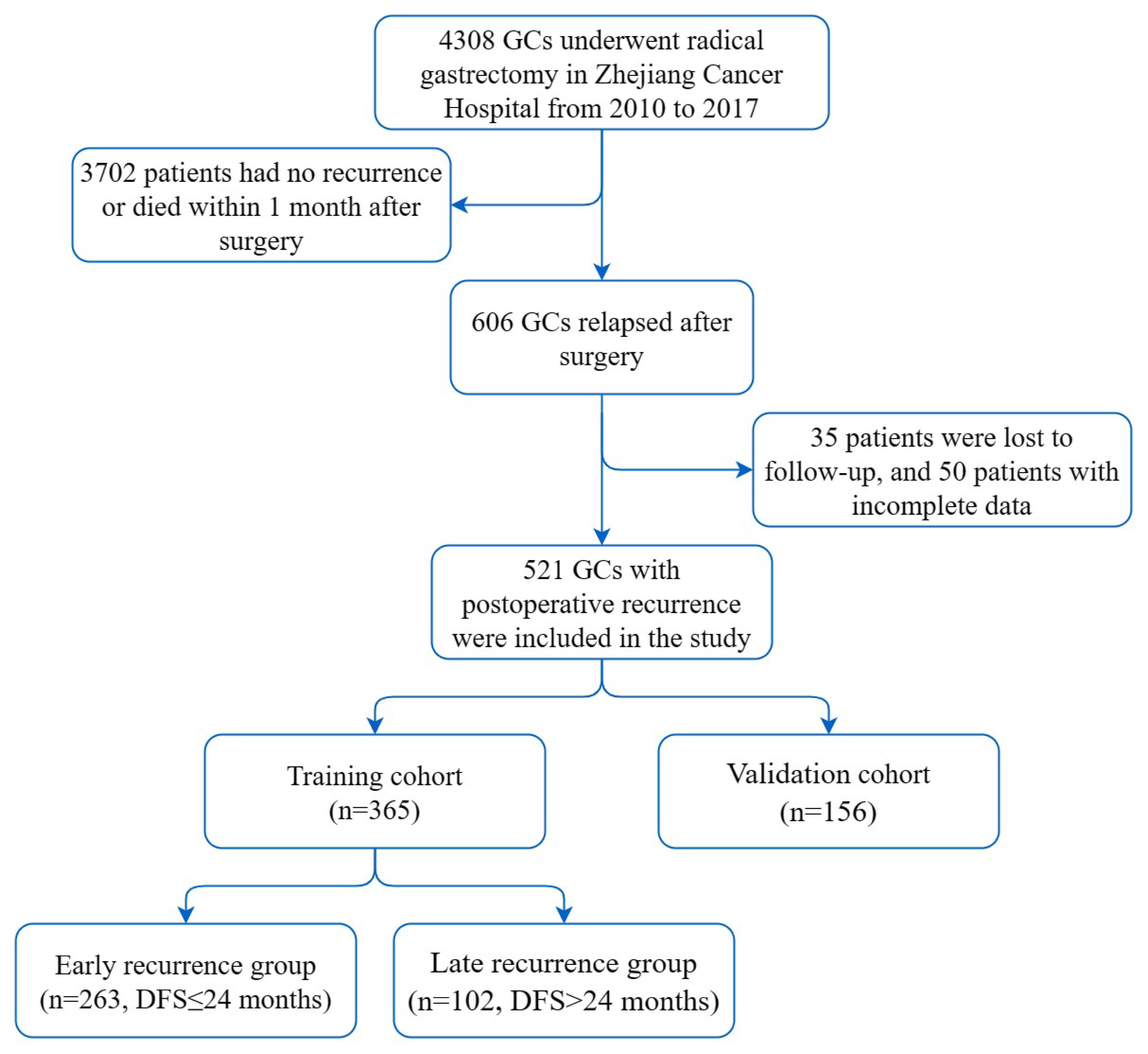
**

**Supplementary Figure S1.** Flow charts for inclusion of patients in training cohort and validation cohort.
